# Zebrafish as a Model for Multiple Sclerosis

**DOI:** 10.3390/biomedicines12102354

**Published:** 2024-10-16

**Authors:** Briana Maktabi, Abigail Collins, Raihaanah Safee, Jada Bouyer, Alexander S. Wisner, Frederick E. Williams, Isaac T. Schiefer

**Affiliations:** 1 Department of Pharmacology and Experimental Therapeutics, University of Toledo, Toledo, OH 43614, USA; 2 Center for Drug Design and Development 3, University of Toledo, Toledo, OH 43614, USA; 3 Department of Pharmacy Practice, University of Toledo, Toledo, OH 43614, USA; 4 Department of Medicinal and Biological Chemistry, University of Toledo, Toledo, OH 43614, USA

**Keywords:** zebrafish, multiple sclerosis, neurodegenerative, in vitro, in vivo

## Abstract

**Background:** Zebrafish have become a key model organism in neuroscience research because of their unique advantages. Their genetic, anatomical, and physiological similarities to humans, coupled with their rapid development and transparent embryos, make them an excellent tool for investigating various aspects of neurobiology. They have specifically emerged as a valuable and versatile model organism in biomedical research, including the study of neurological disorders such as multiple sclerosis. Multiple sclerosis is a chronic autoimmune disease known to cause damage to the myelin sheath that protects the nerves in the brain and spinal cord. **Objective:** This review emphasizes the importance of continued research in both in vitro and in vivo models to advance our understanding of MS and develop effective treatments, ultimately improving the quality of life for those affected by this debilitating disease. **Conclusions:** Recent studies show the significance of zebrafish as a model organism for investigating demyelination and remyelination processes, providing new insights into MS pathology and potential therapies.

## 1. Introduction

### 1.1. Introduction to Zebrafish in Medicine

The use of zebrafish (*Danio Rerio*) to study a variety of biomedical topics is a relatively new technique that has gained recent popularity. The term “zebrafish” appears in over 55,000 publications on PubMed. Forty-three percent of these results were published between 1948 and 2014, while 57% of the results were published in the last 10 years. Such data suggest that using zebrafish for biomedical studies is a recent and efficient way to conduct research.

### 1.2. What Are Zebrafish?

Zebrafish are in the minnow or cyprinid family. They are freshwater organisms that originate from India and southeastern Asia, preferring warm, tropical climates. They consume a varied diet of microorganisms such as larval insects and zooplankton in the wild, or paramecium, rotifers, brine shrimp, and flake food in the lab depending on their life stage [1,2]. Adult male wild-type zebrafish are characterized by a slim body with bright blue/black stripes contrasted against silver stripes. Adult male zebrafish may gain more of a red tint when mating. Adult females are defined by the same blue/black and silver stripes as well as a larger, rounder belly (where eggs are stored). Adult females are less vibrant than their male counterparts. Spawning of embryos occurs in spring conditions. One female may lay over 100 eggs, anticipating that one survives to reproduce in their natural environment [3]. Zebrafish have an average life span of 3 years. They are popular, active fish appreciated by hobbyists and researchers alike.

Zebrafish at the University of Toledo are kept at a temperature of about 25 °C, 7.0 pH, and 500 µs/cm. They follow a light cycle of 14 h of light and 10 h of dark. These conditions set the zebrafish in an eternal spring, encouraging the production and fertilization of eggs for research purposes. In the lab, zebrafish may consume an alternating diet consisting of protein-rich food, like brine shrimp, supplemented with vitamin-rich flake food. Zebrafish may be fed one to three times a day. Adult zebrafish are bred, and eggs are collected weekly. Zebrafish eggs hatch around 3 days post-fertilization (dpf). After 5–7 days of ensuring clean water conditions, larval zebrafish may be used for various research practices. They may also be genetically modified prior to hatching.

### 1.3. Advantages of Zebrafish Models

The use of zebrafish as subjects for research is less common than mice. Mice make up about 95% of all laboratory animals used in biomedical research [4]. This is likely because these mammals share 85% of their protein-coding regions with the human genome [5]. Comparably, zebrafish share about 70% of their genome with humans [4]. What zebrafish lack in genetic similarity to humans, they make up for in their efficient breeding and growth routines. The average female mouse has a gestation of 19–21 dpf, resulting in about 9–12 pups that will not reach adulthood/full development for another 4–7 weeks [4]. Multiple pairs of zebrafish can be bred overnight, producing a clutch of 100–1000 embryos. After 7 dpf, larval zebrafish are developed and ready to be used as subjects. This allows for testing mass amounts of subjects in a short amount of time.

Zebrafish require less space and maintenance and are available at a lower cost. A 3.5 L tank can house about 20 adult zebrafish (1 zebrafish per 1.79 in^2^). A singular mouse weighing over 25 g requires more than 15 in^2^ of space [4]. Zebrafish waste and additional food can be mechanically removed through filters, while mice require manual labor to remove waste. Zebrafish are also cheaper to purchase. The cost of an individual, three-week-old C57BL/6 (a common lab mouse strain) is USD 28.53 on The Jackson Laboratory website (as of July 2024) [6]. According to the Zebrafish International Resource Center (ZIRC) website, a pair of wild-type AB zebrafish cost USD 20.00 (10.00 USD/individual) [7]. These are just a few examples of why zebrafish are more favorable subjects in research compared with mice, specifically in genetic and behavioral studies.

Although using zebrafish as subjects is gaining rapid popularity, it is important to remember that this is a relatively new technique in the scientific community. The science and husbandry of using zebrafish are still being developed and standardized. As mentioned, mammalian subjects, such as mice, make up the majority of lab animals used in biomedical research. This is likely because mice, for example, share more genetic similarities with their human counterparts and their modeling history has been studied and refined for a longer time period in comparison with zebrafish models.

### 1.4. Current Areas of Study and the Future of Zebrafish in Research

Published studies provide evidence that zebrafish have been used to study circadian rhythm, neurotoxicity, ocular health, digestion, auditory health, cardiovascular health, and more [5]. The similarity of the central nervous system (CNS) between zebrafish and mammals is a key factor in the areas of research mentioned above. At the time of testing, it is typical for zebrafish to be 5–7 days old. At the age of 5 days, the zebrafish brain is developed enough to respond to stimuli [8]. In relation to humans, a 5-day-old zebrafish equates to the development of a 6-month-old human infant (a 6-day-old zebrafish is equivalent to a 9-month-old human infant, and a 7-day-old zebrafish to a 1-year-old human infant) [9]. When comparing the brain structure between a zebrafish and a mammal, both contain olfactory bulbs, a habenula, a cerebellum, a medulla, and a spinal cord, among other similarities [10]. It is important to understand the development of the zebrafish CNS in comparison to the human CNS at the time of testing. Studying neurodevelopmental disorders and neurodegenerative diseases in larval zebrafish using high-throughput testing allows for quick results that can be further tested in adult, fully mature zebrafish. This scheme accelerates the pace of research in CNS studies and pharmacology.

**Figure 1:**
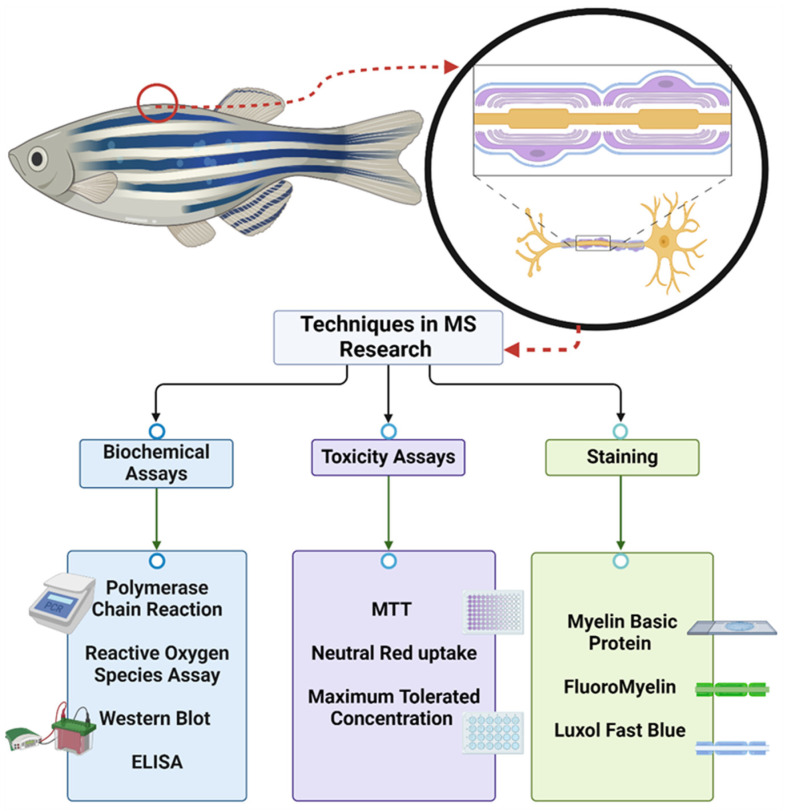
Techniques in MS research. Created with BioRender.com.

Zebrafish have proven to be beneficial models in studying multiple sclerosis (MS) because of their genetic similarity to humans, affordability, low real estate, and efficient breeding and growth rate, among other reasons. Zebrafish have a simple CNS. At the larval stage, their semi-transparent bodies allow for clear imaging of the CNS. Zebrafish can be induced with MS through various processes, including synapse-level analysis of neurocircuitry [11]. After being induced, the zebrafish immune system begins to attack its own myelin sheath through demyelination. The process of demyelination can be observed in real time by using zebrafish as subjects, helping scientists understand MS and test new compounds for therapeutic drug discovery. The root of MS and its remedy are unknown [12]. Studying MS using zebrafish is becoming a more common research practice. Various techniques have been utilized in research, allowing scientists and the general public to understand more about the mysterious disease that is MS (Figure 1).

### 1.5. What Is Multiple Sclerosis?

MS is a chronic autoimmune disease. MS is known to cause damage to the myelin sheath that protects the nerves in the brain and spinal cord. This damage leads to blockage or slowed action of signal transmission across nerves causing adverse neurological effects that can result in a decreased quality of life and disability of the person affected [12,13]. Damage to the myelin sheath is called a lesion, a focal point of demyelination and inflammation on the large network of nerve fibers, also known as white matter [14], that can be seen during magnetic resonance imaging (MRI). After the initial inflammatory phase of MS, the lesions may begin to enter a state of smoldering, where inflammation and demyelination coexist. This state is considered more chronic as remyelination and inflammation resolution without repair occur [15]. Depending on the type of MS a person may have, the state of inflammation and neurodegeneration may vary over time [16].

Damage to the myelin sheath is the primary characteristic of MS. Myelin is an insulating layer made of protein and fatty substances that functions to transmit electrical impulses to nerve cells [17]. More specifically, myelin is made up of oligodendrocytes, the myelinating cells of the CNS. Oligodendrocytes are produced by a series of events including proliferation, migration, differentiation, and myelination that produce the myelin that protects the brain and spinal cord [18]. Without these protective layers, the CNS would be prone to damage and cause a culmination of issues within multiple body systems. Myelin and oligodendrocytes are arguably two of the most important substructures in the human body.

Like other diseases, MS has multiple forms. There are three types of MS, relapsing–remitting (RRMS), primary progressive (PPMS), and secondary progressive (SPMS). RRMS is noted by immune cell-driven plaque-like demyelination with resulting neurodegeneration. The invasion of T cells into the CNS parenchyma is substantially lower than in those with progressive forms of MS [19]. RRMS is the most common, with approximately 85% of cases being this type of MS [20]. PPMS is less common, representing 10 to 20% of all cases, and is noted by steadily worsening symptoms with no state of remission^12^. SPMS is seen as a secondary stage in those with RRMS. Although it may still appear in those undergoing treatment for RRMS, it is more likely to occur in those going untreated, as the treatment plans for RRMS and SPMS are not the same [21]. Like the other forms of MS, SPMS worsens neurological function and increases disability over time. MS typically presents as CIS or clinically isolated syndrome prior to becoming MS [22]. CIS is widely accepted as the precursor to inflammatory CNS diseases, with the conversion rate of CIS to MS being 20 to 75% [23].

MS presents itself in many symptoms, including blurred vision with pain, weakness, paresthesia, impaired sensation in limbs, vertigo, hearing loss, facial sensory issues, and memory loss. MS is diagnosed by a combination of findings discovered by laboratory data and imaging. The Revised McDonald Criteria defines MS as “...the demonstration of dissemination of MS disease characteristics in space and time. Dissemination in space refers to the presence of lesions in distinct CNS anatomical locations including infratentorial, juxtacortical, cortical, periventricular, and spinal cord. Dissemination in time refers to the development of new lesions over time”. MRI is used to monitor the development of MS and in the initial diagnosis of MS [16].

### 1.6. Epidemiology and Etiology of Multiple Sclerosis

MS affects over 2.5 million people worldwide, typically arising between the ages of 20 and 40 years old and affecting women twice as much as men. Although there is no definite cause of MS, genetic and environmental factors may be attributable to its presence. The risk of MS is higher in those with immediate family members who carry it, yielding a 2 to 4% risk of MS development, whereas the general population has a 0.1% chance of developing the disease [24]. It has been found that the HLA-DRB1 allele is one of the highest-risk alleles in MS diagnosis [25]. With genetics only accounting for 30% of MS risk, environmental factors are also considered. Factors such as latitude, low vitamin D, obesity, smoking, and infections have been shown to increase the risk of developing MS [26]. Infectious factors are a large topic of discussion when determining the cause of MS. Epstein–Barr Virus or EBV has been shown to be triggered in genetically predisposed individuals by infectious agents, with EBV being the primary suitor [27]. EBV is a widespread human lymphotropic herpesvirus that has been known to play a role in several cancers, but through recent studies, it has been seen to have a potential role in MS development [27]. Other risk factors for MS development may include age, sex, race, smoking, and obesity [28].

## 2. In Vitro Models

There may not be a “perfect” model that fully replicates the complex nature of MS in vivo or in vitro. However, various models have been established over the years that can imitate specific stages of the disease [29]. Many of these models are also able to replicate the MS pathological process where demyelination, axonal loss, and inflammation are observed [30].

Various in vitro models exist, encompassing both single-cell and mixed-cell cultures (Table 1). MS in vitro models can be divided into immortalized cell lines or isolated mammalian brain cells [31,32]. These cells facilitate studying cell–cell interactions, offering an advantage due to the intricate nature of the CNS. They also facilitate the interactions among CNS cells including immune-neurological interactions, which can be streamlined by using those cells [33]. Microglial cells, astrocytes, neurons, and oligodendrocytes are a few cells used in CNS studies. In this section, some in vitro models used in MS research will be briefly discussed.

### 2.1. Microglia

Microglia activation is observed in various stages of MS lesions [34]. Studies involving microglial cells allow for the examination of how microglia respond to specific signals, including biochemical signals, and the potential mechanisms triggering their activation. Furthermore, a major potential advantage of the use of microglia cultures is their early response to these signals. Cultures are commonly obtained via embryonic or early post-natal animals, which utilized “aged” microglia from adult animals, where cells were obtained postmortem or from surgical material [32,35,36,37]. It is important to note that isolated microglia may not represent the best target for MS research since they are not at the center of the pathophysiology of the disease, as with oligodendrocytes; rather, they react to the existence of the disease, which is difficult to capture in full in vitro. Furthermore, the study of embryonic or early post-natal cell cultures does not mimic the true dynamic and chronic nature of MS. Zebrafish enable the observation of microglial behavior in real time within the living organism, allowing researchers to study their role in neuroinflammation and tissue repair.

### 2.2. Oligodendrocytes

Oligodendrocytes play a vital role in myelin production in the CNS following various developmental stages including pre-progenitor, progenitors, pro-oligodendrocyte, and immature oligodendrocytes. In vivo and in vitro identification of these stages occurs through the expression of different molecules, including myelin proteins such as myelin oligodendrocyte glycoprotein (MOG), which is predominantly found in mature cells [31]. In MS, maintenance and remyelination processes depend upon the proper functioning of oligodendrocytes, involving progenitor migration, differentiation into mature myelin-forming cells, and axon ensheathment. Stimulation of oligodendrocytes in vitro to facilitate migration, differentiation, and ensheathing is essential for developing repair strategies and understanding mechanisms of damage in MS. However, these strategies have shown that remyelination in MS may not precisely replicate developmental myelination processes, emphasizing the role of in vivo models in MS research [31,38].

### 2.3. Astrocytes

While astrocytes play a crucial role in maintaining homeostasis, including preserving the integrity of the BBB, they also contribute to scar tissue formation in chronic MS lesions. In the event of brain damage affecting astrocyte function, they may lose their ability to maintain the BBB, exacerbating further damage. On the other hand, astrocytes are involved in regeneration and aid in the repair process by secreting growth factors. Various human and animal cell lines and primary cultures are utilized to study these functions. Astrocyte cell lines derived from mice, rats, or human astrocytomas [33] have been developed, but they may respond differently than primary astrocytes, which is advantageous in their suitability for repeated passage and cryopreservation [31]. The use of primary cultures, obtained from post-mortem fetal or adult tissues and biopsies from neurosurgery patients that are cleared from any microglial contamination, is considered more reflective of astrocyte responses in the CNS [39,40,41].

### 2.4. Neurons

Primary neuronal cells and cell lines are acquired from embryos of mice, rats, and humans [42,43,44], with recent advances allowing for the generation of neuronal cultures from pluripotent stem cells in rats [45] and rhesus monkeys [46]. Maintaining primary human neurons is challenging because of post-mortem tissue acquisition, but some cells are also induced during surgeries [47,48].

The recognition of axonal damage and neurodegeneration as contributors to MS disease progression has led to the increased use of neuronal cells. Neuronal cell lines address issues like low cell numbers and difficulties in culturing primary neurons. Differentiation strategies have been developed for these cell lines, but challenges persist. Notable cell lines like SH-SY-5Y, HCN [49], and NT2 [50] differentiated with retinoic acid [51,52], or brain-derived growth factor, are commonly employed. However, these cell lines may not fully express mature cell markers and exhibit slow proliferation, necessitating costly growth factors [53]. The need for growth factors is eliminated when employing an in vivo model, such as zebrafish, where rapid neuronal development and myelination are observed as soon as 3 dpf.

### 2.5. Brain Slices and Aggregate Systems

The in vitro models discussed earlier suffer from the following significant drawback: the responses of individual cell types may not fully represent the behavior of these cells within intact tissue, raising questions about their direct relevance to understanding pathogenic or repair mechanisms in MS. Neural cell co-cultures, aggregate cultures, and brain slice (organotypic) preparations maintain intimate contact between CNS cells, potentially better reflecting the human brain microenvironment. Two types of brain slice cultures, i.e., “acute” models and organotypic cultures, have been tailored to study mechanisms relevant to MS. Acute models allow for rapidly conducting studies within hours, while organotypic cultures facilitate more prolonged investigations, including demyelination and remyelination studies.

Brain slice cultures also provide insights into the association between innate immune activation and neurodegeneration in MS [54,55]. While brain slices may provide a more holistic image of the CNS environment in comparison with the previously discussed cell models, they lack the full in vivo environment, including systemic factors and long-range cellular signaling, which can influence pathology and responses to treatment. Additionally, brain slices are typically used for short-term studies, which may not capture the chronic processes involved in MS, such as long-term inflammation or neurodegeneration. Zebrafish, with their live, intact nervous system, offer a superior platform for exploring the multifaceted interactions and processes involved in MS, providing insights into disease mechanisms and potential therapeutic approaches.

After discussing a few types of in vitro models that have been heavily used in research and have shown promising results, it is important to note that researching compounds in vitro is insufficient. In order to investigate the safety and efficacy of these compounds, it is crucial to test them further in vivo.

**Table 1:** In vitro models used in multiple sclerosis-related research.

Cell Type	Cell Line	Derivation	Culture Conditions	Efficacy Readout	References
Microglia	HMO6	Human. Generated by transfection of embryonic human microglia with a retroviral vector containing cDNA encoding for v-myc oncogene.	Maintained in Dulbecco’s modified Eagle medium (DMEM) supplemented with 5% horse serum, 5 mg/mL d-glucose, 25 mg/mL gentamicin, and 2.5 mg/mL amphotericin B (feeding medium).	Immunocytochemistry Fura-2 Ca21-Fluorescence Quantitative Real-Time PCR Analysis Gene Expression of Cytokines and Chemokines ELISA Analysis	[56]
Oligodendrocytes	MO3.13	Human. Fusion of the rhabdomyosarcoma cell line with primary oligodendrocytes [57].	Maintained in Dulbecco’s modified eagle medium supplemented with 10% fetal bovine serum, 2 mM l-glutamine, and 1× penicillin–streptomycin solution.	Immunocytochemistry: Primary antibodies used were NCAM2 and CNPase Cell-based ELISA: Biotinylated anti-human IgG and IgM antibodies were used	[58]
	HOG	Human. Clone derived from oligodendroglioma [59].			
Astrocytes	C6 (rat)	Rat. Derived from a rat glial tumour induced by N-nitrosomethylurea.	Maintained in DMEM containing 10% FCS and antibiotics (100 i.u./mL penicillin and 100 μg/mL streptomycin).	mRNA analysis Luciferase activity assay measurements Glutathione assay Immunoblot analysis Immunocytochemical staining Transcription factor DNA-binding activity assay	[60]
	A172 (human)	Human glioblastoma	Cells were cultivated in α-MEM with glutamine, 10 or 5% fetal calf serum (FCS), 0.1 mg/mL streptomycin, and 100 units penicillin G at 37 °C and 5% CO_2_. Cells were subcultured with trypsin/EDTA every 3–4 days.	Morphology and immunocytochemistry Flow cytometry Quantitative Real-Time PCR Analysis	[61]
	U-87MG (human)	Human glioblastoma	Nicotine was diluted in PBS and added at various concentrations (1, 5, 10, 50, 100, 500, and 1000 μg/well) after a four-hour cell attachment. Cells were incubated for 48 h at 37 °C and then removed from the medium.	MTT assay used for cell viability measurement Evaluation of MMP-2 activity by zymoanalysis	[62]
Neurons	SH-SY5Y	Human. A thrice-cloned sub-line of bone marrow biopsy-derived line SK-N-SH [63].	Maintained in DMEM supplemented with 2 mM L-glutamine 100 units/mL penicillin/streptomycin 1% nonessential amino acids (11140-035, Invitrogen, Paisley, Scotland, U.K.), and 10% (*v*/*v*) heat-inactivated FBS.	MTT assay used for cell viability measurement Determination of Reactive Oxygen Species Immunocytochemistry: Primary antibody used was anti-human Apo D Quantitative Real-Time PCR Analysis	[64]
Brain Slices and Aggregate Systems	N/A	Human	Sixteen coronally cut, 10 mm thick full-hemispheric brain slices of 10 patients with chronic MS were selected at autopsy and were formalin-fixed for several weeks.	Magnetic resonance imaging (MRI) Neuropathology and immunohistochemistry Regional analysis of cortical gray matter Global analysis of cortical gray matter	[65]

## 3. In Vivo Models

### 3.1. Experimental Autoimmune Encephalomyelitis (EAE) Model

The most common model used in MS research is known as the experimental autoimmune encephalomyelitis (EAE) model. EAE has proven valuable in understanding the cascade of events leading to the development of MS.

Unlike the natural sensitization that occurs in humans with MS, EAE is induced in animals such as rodents and monkeys via an external immunization step [66]. In EAE, sensitization to myelin antigens is typically achieved using adjuvants, which contain bacterial components that activate the innate immune system [67]. This immunization step helps researchers identify and control inducing antigens in contrast to MS, where no unique antigen has been identified [68]. Given its ability to activate the immune and nervous systems, the EAE model can replicate the fundamental characteristics of MS. In addition to its utilization in capturing the pathophysiology, it has also been employed in treatment testing and development. Glatiramer acetate and natalizumab are examples of MS medications that were tested in preclinical studies on the EAE model [69,70].

Despite the EAE model’s fruitful mirroring of the pathophysiology of MS, discrepancies from a therapeutic perspective exist, as treatments that have shown success in an EAE model may not necessarily have a positive effect in MS [71]. Furthermore, a significant disadvantage of this model is its inability to detect certain side effects of treatments that humans are susceptible to in comparison with animals. An important example is the reactivation of latent John Cunningham virus (JCV) leading to progressive multifocal leukoencephalopathy (PML) with medications such as natalizumab, which did not occur in EAE experiments [72]. This highlights the importance of adapting even the most successful experimental models to the particular clinical or scientific question under examination.

### 3.2. Chemically Induced Model

Chemically induced models of MS provide valuable tools for investigating the complex mechanisms underlying demyelination and remyelination processes. The three commonly used chemically induced MS models include cuprizone-induced demyelination, lysolecithin-induced demyelination, and ethidium bromide-induced demyelination. Each model offers unique advantages, allowing researchers to study different aspects of MS pathology and evaluate potential therapeutic interventions.

Cuprizone, a copper-chelating agent, is utilized in the systemic induction of CNS demyelination, replicating the demyelination process that occurs in MS [73]. This occurs due to cuprizone’s effect on damaging oligodendrocytes, leading to activation of the microglia and mitochondrial dysfunction [74,75]. A major advantage of the cuprizone model is the shift to a remyelination process that becomes possible when cuprizone administration is halted, which is not possible with EAE. While inflammation is a key feature of MS, disease progression and therefore disability are driven by the failure to remyelinate [76]. Therefore, the cuprizone model not only allows for studying both the demyelination and remyelination processes within the same model, but it also allows for investigating and evaluating potential remyelination therapies. However, it is important to note that various factors such as animal species, weight, age, genetic background, and means of cuprizone administration can alter the response to this agent and, therefore, the results expressed by the model [77,78,79,80].

Lysophosphatidylcholine (LPC), or lysolecithin, is another toxin-induced demyelinating model used in animals including mice, rats, rabbits, and cats. LPC injections at a concentration of 1% adequately create lesions in the spinal cord and the corpus callosum by binding to and disrupting myelin’s lipid structure [81,82,83,84,85,86,87]. As with the cuprizone model, demyelination is followed by a remyelination phase, which may be seen as soon as 7 and 10 days post-injection in rodents [88,89].

Ethidium bromide interacts with DNA and primarily targets astrocytes, leading to the induction of demyelinating lesions due to the lack of support factors released by these cells. This model has illustrated the critical role of astrocytes in oligodendrocyte-mediated remyelination. Understanding the interactions between astrocytes and oligodendrocytes in the context of remyelination offers insights into potential therapeutic strategies for MS [90,91].

While these toxin-based models share the attraction of rapid remyelination with toxin discontinuation, they lack the adaptive immune system or inflammatory characteristics of MS since demyelination is triggered independent of lymphocyte activation [92,93]. On the other hand, activation of the immune system and replication of the inflammatory processes of MS in EAE leads to severe demyelination preventing possible remyelination [94]. To address these challenges, combination models have also been developed in an effort to combine the study of remyelination in the presence of inflammatory processes [95].

### 3.3. Zebrafish Models of MS

The zebrafish is a small, optically transparent animal with rapid embryonic development, allowing for real-time analysis of myelination and remyelination processes. Myelinated axons can be observed from the third day post-fertilization, providing an efficient platform for in vivo studies [96,97]. Additionally, zebrafish are distinguished by their high fecundity and genetic tractability and are more economical to maintain than other experimental animals. These advantages, coupled with the ability to create transgenic lines expressing green fluorescent protein in oligodendrocytes and precursor cells, facilitate the investigation of remyelination mechanisms and the investigation of potential treatments promoting myelin repair [98,99,100,101].

Furthermore, the zebrafish model offers unique opportunities for studying in vivo vertebrate myelination. Rapid myelination occurs within the first week of zebrafish life, impressively shortening the time needed for research compared with rodents. Ultrastructure analysis has demonstrated the similarity in myelin between zebrafish and mammals, endorsing zebrafish as a model for investigating conserved molecular mechanisms promoting myelination. Advances in gene manipulation techniques enable precise molecular alterations in developing zebrafish embryos, facilitating the study of target genes and molecules. Comprehensive mutational and pharmacological testing can be efficiently conducted because of the high number of offspring produced in a single mating and the ex-utero development of zebrafish embryos, offering promising avenues for identifying novel molecules and genes involved in vertebrate myelination [96,102].

## 4. Studies

Zebrafish have become a key model organism in neuroscience research because of their unique advantages. Liu et al. [103] leveraged these features to investigate the effects of cuprizone-induced demyelination on dopaminergic hyperactivity and locomotor deficits in zebrafish larvae. Exposing zebrafish larvae to cuprizone resulted in significant behavioral and physiological changes, including reduced overall locomotor activity and diminished responses to acoustic and light stimuli. These effects were associated with the upregulation of several dopamine receptor genes, highlighting the role of dopaminergic hyperactivity in the functional processes. Although their study provided valuable insights into the negative impact of demyelination, it did not assess the behavioral or physiological effects of the zebrafish after potential remyelination. Future research could use combinecuprizone-induced demyelination with drugs targeting remyelination to evaluate their impact on dopaminergic activity in zebrafish. This research underscores the utility of zebrafish as a model for studying the neurobiological effects of demyelinating insults [103].

Zhu et al. [104] developed a zebrafish demyelination model using ethidium bromide (EB), a gliotoxin-like agent that induces demyelination by killing oligodendroglial, astrocytic, and Schwann cells. They optimized the EB concentration and treatment duration, finding that 75 μM EB for 72 h effectively induced demyelination and decreased motility in a dose-dependent manner. After EB removal, treatment with thyroxine (T4) promoted remyelination and improved motility, confirming T4’s efficacy in myelin repair. Additionally, RAR-related orphan receptor-γt (ROR-γt) inhibitors GSK805 and SR1001 enhanced remyelination and facilitated regeneration of myelin sheath components and axons, with SR1001 showing higher recovery rates at increased concentrations. These findings suggest that ROR-γt inhibitors and T4 are potential therapeutic agents for demyelinating disorders and that the zebrafish model provides a reliable method for screening remyelination compounds and evaluating potential treatments for MS symptoms.

In a study by Kim et al. [105], the potential of sulfasalazine to promote remyelination in zebrafish was investigated. Sulfasalazine commonly used to treat inflammatory bowel disease and rheumatoid arthritis, has shown neuroprotective effects. Using a transgenic zebrafish model containing the bacterial cytotoxin gene nfsB, which converts prodrugs like metronidazole into cytotoxins, they induced rapid ablation of olgiodendrocytes and Schwann cells upon metronidazole exposure, resulting in CNS demyelination. Following demyelination, sulfasalazine treatment encouraged remyelination, evidenced by the recovery of oligodendrocytes and Schwann cells, visible myelin sheaths, and low g-ratio values. Sulfasalazine also decreased macrophage/microglia in demyelinated brains; while these cells are essential for initiating remyelination, their prolonged presence can hinder the process. Their study demonstrates sulfasalazine as a promoter of remyelination in zebrafish and highlights the importance of macrophage/microglia dynamics during demyelination and remyelination stages. However, the complete ablation of oligodendrocytes and Schwann cells may not fully translate to clinical scenarios of MS. Future studies might develop a transgenic zebrafish model allowing controlled ablation of these cells. Overall, these findings suggest sulfasalazine’s potential as a therapeutic agent for demyelinating disorders [105].

Münzel et al. [106] employed zebrafish to investigate optic nerve regeneration after demyelination. They developed a zebrafish model using lysophosphatidylcholine (LPC) to induce demyelination, assessing remyelination and axonal damage in both young and old zebrafish. They found that while zebrafish can fully regenerate optic nerve myelin post-demyelination, this ability diminishes with age, suggesting age-related changes in remyelination processes. Applying LPC onto gelatin foam induced demyelination, which peaked on day 3 and recovered by day 28. Markers such as Claudin k, Olig2-positive oligodendroglial cells, and 4C4-positive microglia/macrophages normalized by 28 days. Remyelination in young adult zebrafish showed that at 8 days post-lesion, 24% of axons were myelinated compared with the controls (93%), but by 28 days, the percentage matched the controls. Older zebrafish exhibited comparable remyelination rates but thinner myelin sheaths, persisting at 3 months, indicating age-related myelin decline. While the number of microglia and macrophages did not differ between young and old demyelinated zebrafish, fewer were present at the lesion site in older zebrafish 4 days post-lesion, suggesting inadequate recruitment potentially impacting remyelination. Their study highlights zebrafish as a model for investigating demyelination and remyelination processes, uncovers age-related differences in myelin quality and immune response dynamics, and advances our understanding of regenerative capacities and potential therapeutic targets.

A research article by Buckley et al. [107] investigated the temporal dynamics of myelination in the zebrafish spinal cord and assessed the impact of drugs on remyelination They characterized the spatiotemporal patterns of myelination in zebrafish larvae, identifying key developmental stages in myelin formation. Their study highlights targeting endogenous remyelination as an alternative approach for diseases like MS, emphasizing the need to manipulate oligodendrocyte precursor cell differentiation to promote remyelination. The structural properties and cell lineage of oligodendrocytes are highly comparable between zebrafish and mammals, although the expression patterns of major myelin-associated genes vary. Current methods are developing transgenic zebrafish models that can engage in targeted gene deactivation. Future research could focus on overcoming the limitation of differences in gene expression patterns to enhance the relevance of zebrafish models in myelination research. By elucidating the mechanisms governing myelination in zebrafish, this research contributes to understanding nervous system development and function. Collectively, these and other studies (Table 2) call attention to the versatility of zebrafish as a model organism for investigating demyelination, remyelination, and associated behavioral deficits. By leveraging the unique advantages of the zebrafish model, researchers can gain a better understanding of the underlying mechanisms and identify potential therapeutic targets for treating demyelinating disorders.

## 5. Conclusions

Zebrafish have emerged as a valuable and versatile model organism in biomedical research, including the study of neurological disorders [109]. Their genetic, anatomical, and physiological similarities to humans, coupled with their rapid development and transparent embryos, make them an excellent tool for investigating various aspects of neurobiology [109,110]. When it comes to modeling neurological disorders, zebrafish have demonstrated relevance and utility, including their potential for studying demyelination and neuroinflammation [111].

Zebrafish models have also been proven valuable for drug discovery and therapeutic development. Compounds that show promising effects in zebrafish models of demyelination or neuroinflammation can be further investigated in more complex mammalian systems, potentially accelerating the drug development process.

Moreover, while the application of zebrafish in research is relatively new, the growing body of literature accentuates their potential across various biomedical fields. As methodologies and ethical standards for zebrafish research are refined, the scientific community can expect to uncover further insights into human diseases, ultimately contributing to improved health outcomes. While the scientific community continues to refine zebrafish models and explore their applications across diverse research areas, further advancements in understanding disease mechanisms and developing effective treatments are on the horizon. Thus, the future of zebrafish in medicine appears promising, heralding a new era of discovery that could transform our approach to understanding and treating chronic conditions like MS.

## Figures and Tables

**Table 2:** Zebrafish models used in multiple sclerosis-related research.

Strain	Fish Mode	Fish Age	Behavioral Assessment (Yes/No)	Efficacy Readout	Findings	References
Wild-type zebrafish (Danio rerio)	EAE	4–6-month-old fish	No	Optimization of immunization dose Histopathological evaluation used for validation of the model	The EAE model was developed by disease induction with myelin oligodendrocyte glycoprotein (0.6 mg/mL of MOG), model validated using fingolimod	[108]
Wild type	Chemically Induced: Cuprizone	Larvae Cuprizone exposure: 6–8 h post-fertilization (hpf) Behavioral Analysis: 120 hpf	Yes, post-demyelination	Automated video-tracking system used for behavioral analysis Neurotransmitter measurement RNA-seq and bioinformatic analysis Quantitative Real-Time PCR Analysis Whole mount in situ hybridization	Cuprizone reduced overall locomotor activity and diminished responses to acoustic and light stimuli; effects were associated with the upregulation of several dopamine receptor genes	[103,106]
Wild-type (WIK) tg (olig2:DsRed) tg (claudink:GFP) tg (claudink: GFP/olig2:DsRed) tg (FoxD3:GFP)	Chemically Induced: Lysophosphatidylcholine	4–7 months young adult 15–18 months aged adult	No	Axonal tracing Tissue processing and immunohistochemistry Electron microscopy	Applying LPC onto gelatin foam induced demyelination, which peaked at day 3 and recovered by day 28 Zebrafish regenerate optic nerve myelin post-demyelination; this ability diminishes with age, suggesting age-related changes in remyelination processes	
Wild-type AB line Green-fluorescent-protein transgenic zebrafish Neutrophil green-fluorescent-protein transgenic zebrafish	Chemically Induced: Ethidium Bromide	Larvae 2–6 dpf	Yes	Determination of no observed adverse effect level (NOAEL) Zebrafish demyelination model validation: Motility assay and FluoroMyelin staining and whole mount anti-MBP immunostaining for demyelination model validation Compound effect assessments: Dose-response assay of remyelination, whole mount anti-acetylated tubulin immunostaining, promotion of peripheral motor neuron, reduction in neutrophil infiltration, and reduction in macrophage recruitment Video-track motion detector used for motility assay	75 μM EB for 72 h effectively induced demyelination, decreased motility Thyroxine (T4) promoted remyelination and improved motility	[104]

## Data Availability

No new data were created or analyzed in this study. Data sharing is not applicable to this article.
